# Just What the Doctor Ordered: Using Parks to Improve Children’s Health

**DOI:** 10.1289/ehp.123-A254

**Published:** 2015-10-01

**Authors:** Nate Seltenrich

**Affiliations:** Nate Seltenrich covers science and the environment from Petaluma, CA. His work has appeared in *High Country News*, *Sierra*, *Yale Environment 360*, *Earth Island Journal*, and other regional and national publications.

For children today, time spent outdoors is becoming more of a luxury—or in some cases, a chore—than a staple. In recent years “nature deficit disorder” among kids has evolved from a turn of phrase^1^ to a cultural indictment.^2^^,^^3^ Smartphones and other screens are increasingly vying for kids’ attention,^4^ but blame lies elsewhere, too: just as recess is being reduced or phased out in many schools, children’s activities are being increasingly structured and scheduled, and concerns over neighborhood crime and safety can impede their ability to play freely outdoors.^5^ A 2013 study by the U.S. Centers for Disease Control and Prevention found that nearly three-quarters of high-school students had less than one hour of physical activity per day,^6^ while childhood obesity rates are trending steadily upward.^7^

**Figure:**
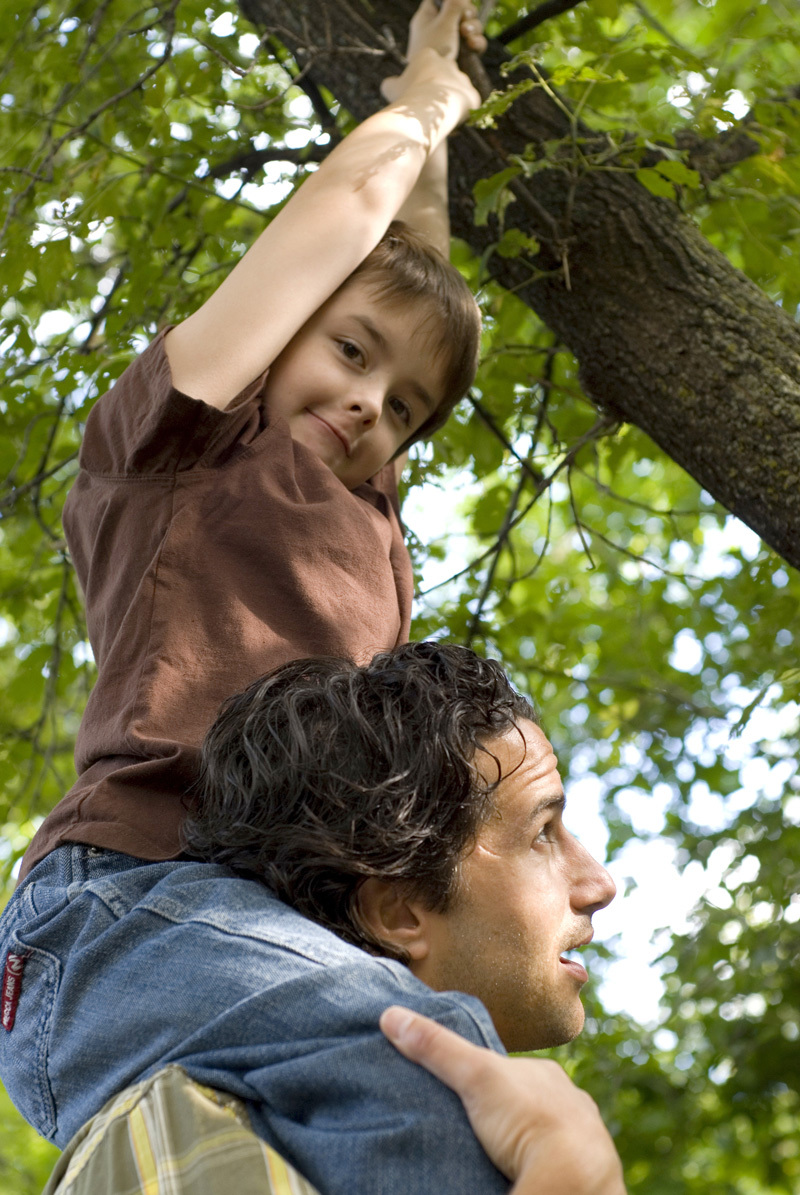
“Park prescriptions” are gaining popularity as researchers learn more about the benefits of spending time in nature. There’s more to learn, however, about the ways in which nature imparts these benefits and the “dose” of nature required to achieve them. © Wave Royalty Free/Photo Researchers, Inc.

Almost at the same time, researchers have dramatically expanded their understanding of the positive link between health and parks of all sorts—from the most majestic national parks to regional community parks and urban “pocket parks” with just a swing set or a few benches. They have also begun to disentangle some of the many pathways through which these benefits appear to occur.^8^^,^^9^^,^^10^^,^^11^^,^^12^^,^^13^ That knowledge is giving rise to a nationwide movement to integrate park visits into disease treatment and prevention through “park prescription” programs.^14^

In some cases, the programs have received support from the Healthy Parks Healthy People initiative of the National Park Service (NPS), launched as a pilot in 2011 and set to expand significantly in 2016 and beyond.^15^ The Healthy Parks Healthy People program aims to improve health through regular use and enjoyment of parks and public lands nationwide, says Sara Newman, director of the NPS Office of Public Health. The NPS is also participating in the White House’s Every Kid in a Park initiative, launched in September 2015. This program provides fourth-graders and their families free admission to national parks and other federal lands and waters for a full year, and transportation support to schools that need it.^16^

Other park-prescription programs are locally managed and operated, including a growing number organized around urban trails and other nontraditional “linear” parks that promote nonmotorized transport.

Current evidence suggests that children have much to gain from time spent outdoors and much to lose from a lack of park access.^17^^,^^18^ In addition to myriad health benefits offered by physical activity in general, research has shown that outdoor exercise in nature can enhance emotional well-being and amplify the benefits of physical exercise.^19^^,^^20^ And for kids in particular, being in or near green spaces has been found to be associated with better test scores,^21^ improved self-discipline^22^ and cognition,^23^^,^^24^ and reduced behavioral problems and symptoms of attention-deficit/hyperactivity disorder (ADHD).^25^^,^^26^^,^^27^^,^^28^

Several studies suggest that spending time outdoors can protect against myopia (nearsightedness).^29^^,^^30^^,^^31^^,^^32^ Still other research indicates that neighborhood green space may help mitigate income-related health disparities.^33^

Parks and green spaces also may contribute to population health by reducing exposures to air pollution^34^ and noise,^35^ capturing and filtering stormwater runoff,^36^ and mitigating heat-related illnesses.^37^ These benefits make urban parks an important tool in climate change adaptation, says Payam Dadvand, a researcher and assistant professor at the Centre for Research in Environmental Epidemiology (CREAL) in Barcelona.

Eventually, current trends could lead parks straight to the core of the American healthcare system, says Kristin Wheeler, associate director of the Institute at the Golden Gate, a nonprofit partner of the NPS.^38^ Someday patients could be incentivized through their insurance to be active outdoors, she suggests, similar to existing small-scale programs that offer financial benefits for exercise and other healthy activities.^39^^,^^40^

“This is the way that nature and parks are going to be talked about—it’s going to be commonplace for your doctor to ask you about how much time you’ve spent in nature,” Wheeler says. “It’s this perfect culmination, where everybody’s starting to see this as the wave of the future.”

## Let’s Go to the Park!

Zarnaaz Bashir, director of strategic health initiatives for the nonprofit National Recreation and Park Association,^41^ is riding the crest of that wave. Her job is to track and support the park-prescription movement within local parks and recreation agencies nationwide, a task that’s becoming increasingly demanding. “People are realizing that we need to find easier ways to get people healthy, and parks are part of the solution,” she says. “I get at least one or two inquiries a week, anywhere from ‘How do I start doing this?’ to ‘How do I get my physicians on board?’ It is moving very quickly.”

At least 75–100 distinct “park prescription” efforts already exist nationwide, according to Bashir. She says the programs, most of which are just one or two years old, vary in terms of how successful and how intensive they are. In any case, for the concept to truly stick and to mature beyond small-scale local initiatives, research needs to follow apace. “We’re looking for evidence that shows that the whole process of prescribing wellness at a park to a patient and going through a specific intervention is working and providing physical and mental health benefits,” she says.

Nooshin Razani, a clinical scientist and pediatrician at UCSF Benioff Children’s Hospital Oakland (UBCHO), is part of a team undertaking a study to provide just such validation. Building off a park visitation program that UBCHO Primary Care Clinic launched with a regional parks district in May 2014,^42^^,^^43^ the study will evaluate the impact of group outings for families in a low-income clinic population. Participants and their families will receive transportation to parks, where they will engage in community-building activities and physical exercise. Investigators will follow participants’ health outcomes, enjoyment of nature, and park visitation practices.

“This is a pilot study based on our experiences going into nature with patients,” says Razani. “We observed that when we spent time in parks with families, children expressed joy and caregivers expressed that they were relaxed and finding new friends. There is this potential that nature can serve as a low-cost, readily available tool for buffering stress. However, since there are few resources in our clinic and many competing needs in the communities we serve, we feel compelled to find the evidence for parks as a health intervention before making assumptions.”

In addition to quantifying health benefits associated with specific interventions, researchers must also identify the mechanisms through which they occur. It’s possible that mental health is a sort of “common pathway” through which most benefits of parks and green spaces travel, suggests Peter James, a research fellow at Harvard University. In other words, mental health may not be solely an end point but also a mediator for benefits related to chronic disease and other elements of overall health.

More specifically, Dadvand notes two of the primary avenues through which green spaces likely promote better physical and mental health. The first is nature’s mentally restorative quality. The second is the social cohesion—the willingness of members of a group to cooperate for the common good^44^—gained through spending time outdoors with friends, family, neighbors, and even strangers.^45^

Originally described in the late 1980s, attention restoration theory holds that many of the benefits of being in nature are related to its ability to rest and restore “directed attention” (focus and concentration) while gently engaging “involuntary attention” (a more relaxed state open to passing stimuli).^46^ Nancy Wells, an associate professor at Cornell University whose work has been informed by this theory, agrees that cognitive mechanisms could account for a wide range of outcomes. “Having better cognitive function is likely to help you eat healthy and help you have a good physical activity regimen, because it affects how well you can cope and how well you’re managing life,” she explains.

Frances “Ming” Kuo, a researcher and associate professor at the University of Illinois at Urbana–Champaign, recently published a review that discussed 21 mechanisms for which there is evidence of a role in the nature–health connection.^47^ She divided the mechanisms into three groups. So-called active ingredients—or specific environmental conditions—include volatile organic chemicals emitted by plants, the sights and sounds of nature, and protection from air pollutants and heat. Physiological and psychological states include increased levels of health-protective factors in the body. Finally, behaviors and conditions include improved sleep and reduced obesity. Among the different mechanisms, Kuo found a common thread that she speculates is central to nature’s health benefits: enhanced immune function. She describes nature, with its many mechanisms of action, as a “multivitamin” providing numerous potentially protective “nutrients” in a single dose.

Physical activity in parks remains a rich area of study, with significant implications for individual fitness as well as community-scale urban planning.^48^ Michael Jerrett, director of the Center for Occupational and Environmental Health at the University of California, Los Angeles, reported in 2012 that access to green spaces was correlated with higher levels of physical activity among youth.^49^ “What we were able to show is that minute by minute, when children went into a green area, their odds of being moderately or vigorously physically active went up by about forty percent,” he says. “Our research also shows that when planners design communities smartly with safe routes for walking to green space near homes, children will generally become more active.”

Other studies have suggested that the relationship between parks and physical activity is more complex, hinging on a variety of socioeconomic factors and park characteristics.^50^^,^^51^ And some have reported no relationship at all, which a recent review posits may be due in part to methodological issues.^52^

Deborah Cohen, a senior natural scientist with the RAND Corporation in Southern California, has sought to understand how specific park features influence physical activity. Results to date indicate that programming and activities are particularly good at drawing users to a park, and that elements appealing to different age groups and physical capabilities are also important.^53^^,^^54^^,^^55^ On the other hand, safety concerns can be a significant impediment to physical activity in parks, particularly for children.^56^

Cohen’s team is currently assessing park use and physical activity in 174 urban neighborhood parks in 25 states. Their goal is to better understand what gets people into parks and how to improve parks so they attract people. “Just an empty piece of land isn’t going to take someone away from an exciting movie or television show or computer game,” Cohen says. “If we want people to be active and stay healthy, we have to compete with those sort of things.”

Proponents of park prescriptions recognize the challenge. “The battle is just getting over that hurdle of connecting to the outside of our home in a safe, happy, healthy way,” says UBCHO’s Razani. “I feel that we must attach more people to outdoor spaces because it’s in a crisis situation.”

**Figure:**
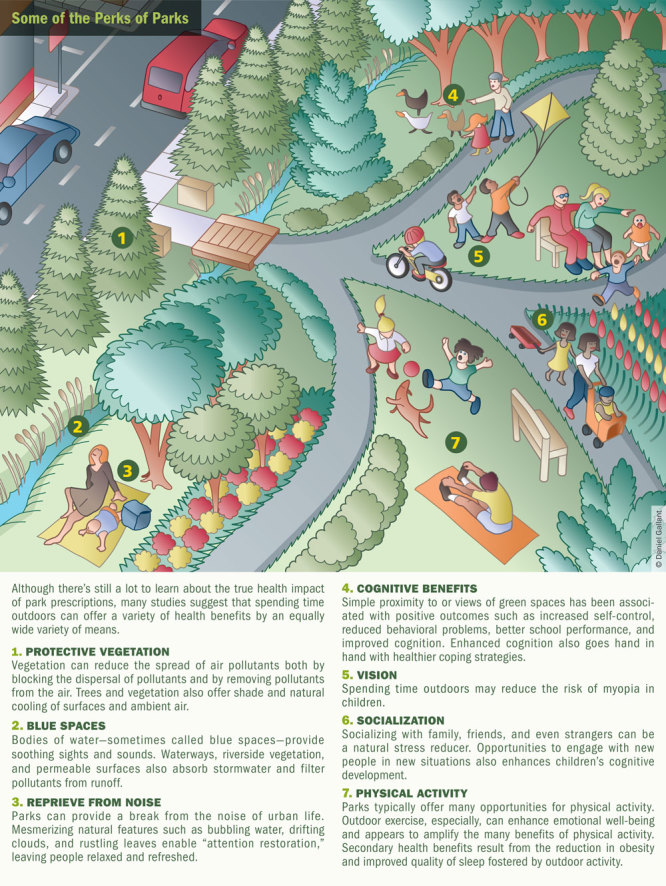


## Getting Doctors Onboard

Healthy Parks Healthy People Bay Area, which Razani has played a role in shaping, is the nation’s most comprehensive park-prescription program.^57^^,^^58^ Launched in 2012, the effort involves numerous partners in all nine counties of the 7.1 million-resident metro area. It supports weekly, biweekly, and monthly activities targeting physical and mental health in a total of 35 parks.

In addition, the City of San Francisco is, to date, the only city in the world to have fully adopted park prescriptions within its Department of Public Health, with weekly health-oriented programs at parks throughout the city.^59^ The program is still in pilot mode, says Curtis Chan, deputy health officer and medical director of the department’s Maternal, Child, and Adolescent Health Section. Chan notes the program has had to combat some inertia in getting physicians to prescribe parks, but critical partnerships are being cemented citywide.

On the opposite coast, Washington, DC, is home to another well-known park-prescription program. There, Unity Health Care pediatrician and park-prescription advocate Robert Zarr has built a program from the ground up in which he has mapped and rated all the green spaces in the city for accessibility, cleanliness, safety, amenities, and services, producing a database that can be linked directly to patients’ electronic medical records. Zarr’s program has considerably less institutional support than the Bay Area program^60^ and therefore lacks the former’s broad scope and participation. But it has still served as a model for other park-prescription programs across the country.

Currently 180 doctors at 26 Unity Health Care locations across the city have signed on, representing a potential reach of more than 100,000 patients. “If I’m successful in the next year and a half, I should have almost every pediatric practice [in the city] on board with prescribing nature,” Zarr says.

Kids in Zarr’s own clinic, which serves low-income and uninsured families (like all of Unity Health Care), have high rates of obesity, asthma, and mental health disorders including ADHD, depression, and anxiety. They also rarely visit parks, consistent with broader findings about socioeconomic factors and access to green spaces.^61^ “I have many, many patients who spend little to no time outside,” he says.

While Zarr has yet to formally evaluate the health effects of his park referrals, he recently performed a study of 212 patients, currently under review at the *Journal of Physical Activity and Health*, that he says shows a statistically significant increase in time spent outdoors following a prescription.

Gilbert Liu, a pediatrician and associate professor at the University of Louisville, recently completed a 20-month pilot park-prescription program of his own and a concurrent study to determine if park referrals changed patients’ behavior.^62^ Like Zarr, Liu developed a GIS-based map that could interface with patients’ medical records and automatically provide a list of nearby parks and other resources such as dance studios and martial arts dojos. Unlike Zarr, however, Liu’s unpublished findings showed that pediatricians’ referrals to visit parks were ineffective. The system was well received by providers, he says, but it turns out that families didn’t use community resources or change their behaviors any more than a random sample of families who didn’t use the system.

“It’s clear that you can’t just tell somebody, ‘Hey, you really need to go to a nearby park and exercise more,’” Liu says. “You need to figure out what exercise is attractive to patients, then identify barriers and overcome those.”

Prescription Trails, based in Albuquerque, New Mexico, and likely the nation’s longest-running park-prescription program, has since 2008 refined and expanded its own approach to encouraging physical activity. Detailed park descriptions and an electronic, customizable “prescription” serve as tools for physicians and other healthcare professionals to encourage patients to be active outdoors.^63^

While the New Mexico program’s grassroots approach has inspired similar efforts in other cities and states, executive director Charmaine Lindblad says she hasn’t yet found funding to assess how many local physicians have embraced park prescriptions, let alone health outcomes for the patients of those who have. “It kind of just exploded, and we’ve never been able to really keep track of who’s using it,” she says.

**Figure:**
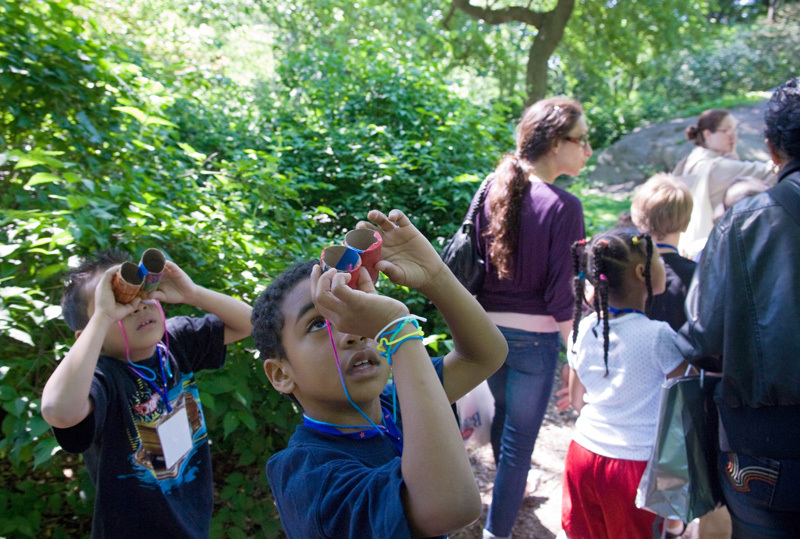
These students made their own binoculars to go birdwatching in New York’s Central Park. At 843 acres, Central Park provides a dazzling variety of settings for kids (and adults) to enjoy nature. But even small, simple green spaces, such as tiny pocket parks tucked between city buildings, can offer health benefits. © Frances Roberts/Alamy Stock Photo

## Expanding the Concept

In the coming months, park-prescription proponents will collaborate on establishing a new framework for regional park–health partnerships nationwide. “A lot of these programs have been built on the backs of individuals, but right now we are working on a larger-scale program,” Wheeler says.

In April 2016 Wheeler, Bashir, and others will organize the nation’s first-ever conference on park prescriptions, designed to provide practical information for implementing and carrying out local programs. For the last two years Bashir and Wheeler have organized a smaller summit on the subject for some of its leaders nationwide,^64^ and in September 2015 Dadvand chaired a symposium on green spaces and health at the annual conference of the International Society for Environmental Epidemiology.^65^

With the Bay Area program as a model, the NPS hopes to bring the Healthy Parks Healthy People concept to communities across the country. “We are at the tail end of that first phase of this program, and now what we want to do in the National Park Service is implement some of the best practices and build and sustain a national model,” says Newman.

One beneficiary of emerging science and the first wave of implementation is Dorothy Ibes, a lecturer and director of the Parks Research Lab at the College of William and Mary in Williamsburg, Virginia. Ibes is currently piloting a new park-prescription program—GWA Park Rx (Greater Williamsburg Area Park Prescriptions)—with a group of physicians and psychologists on campus and in the community.^66^ Ibes’ program is heavily science-based, informed by an exhaustive in-house review of literature on the connection between parks and health and an analysis of existing park-prescription programs.

Once it is widely established, GWA Park Rx will serve as a laboratory for Ibes’ research on the mental and physical health outcomes of park prescriptions as well as on ways to optimize park-prescription programs more broadly. “It’s really about getting your dose of nature right where you are and integrating it into your daily activities,” she says.

Programs like hers could translate to more opportunities for the randomized controlled studies that most researchers in the field say are needed. Without rigorous longitudinal or prospective studies to establish causality and dose–response metrics, says CREAL’s Dadvand, researchers won’t be able to provide the sort of results that many healthcare providers, insurers, and government agencies require to fully buy in to the park-prescription concept.

“We have a tremendous amount of evidence tying access to nature to better health outcomes for a staggering array of specific diseases and disease categories,” says Kuo. “But you don’t know for sure that there isn’t something else going on until you actually do a randomized trial. Until you’ve ruled out those other possibilities, then as scientists you can never say for sure.”

While leadership from the healthcare sector on the national scale has been largely absent to date, Kaiser Permanente, one of the country’s largest providers with more than 10 million members,^67^ is invested in parks as a prevention tool. The company spent more than $11 million over the last seven years to improve access to green spaces nationwide, says Ray Baxter, senior vice president of community benefit, research, and health policy. While Kaiser Permanente doesn’t direct its doctors to provide park prescriptions or have any plans to do so, it is exploring the idea of developing a resource locator for referring patients to appropriate local parks, Baxter says.

Wheeler acknowledges that practical roadblocks exist to full acceptance within the healthcare industry. These include finding a way for providers to bill their time in prescribing parks and, as Liu and others have found, learning how to change the behavior of patients who may be more inclined to take a pill than to visit a park.

Perhaps the key is embracing a shift in perspective from treatment to prevention, and from negative to positive environmental health factors, James suggests.^68^ “We’ve been so focused on air pollution and other harmful exposures, but this is an ‘exposure’ that we can really see as a health benefit,” he says. “This is kind of a win–win where there do seem to be a lot of upsides, and I think that’s what makes this really exciting. It’s just a really nice intervention with a lot of hope.”

**Learn more about the programs mentioned in this story**

**DC Park Rx**

American Academy of Pediatrics District of Columbia Chapter and partners

http://aapdc.org/chapter-initiatives/dc-park-rx/

**Every Kid in a Park**

U.S. Department of the Interior

https://everykidinapark.gov/

**Greater Williamsburg Area Park Prescriptions**

Parks Research Lab, College of William and Mary

http://www.gwaparkrx.com/

**Healthy Parks Healthy People**

National Park Service

http://www.nps.gov/public_health/hp/hphp.htm

**Healthy Parks Healthy People Bay Area**

Institute at the Golden Gate and partners

http://instituteatgoldengate.org/hphpbayarea

**Healthy Parks Healthy People San Francisco**

SF Rec and Park in partnership with Healthy Parks Healthy People Bay Area

http://sfrecpark.org/recprogram/healthy-parks-healthy-people/

**Prescribing Parks for Better Health: Success Stories**

National Recreation and Park Association

http://www.nrpa.org/Grants-and-Partners/Recreation-and-Health/Park-Prescriptions/

**Prescription Trails**

New Mexico Health Care Takes on Diabetes

http://prescriptiontrails.org/index/index.shtml

**Stay Healthy In Nature Every day (SHINE)**

East Bay Regional Park District and UCSF Benioff Children’s Hospital Oakland

http://www.ebparks.org/activities/hphp/shine

## References

[1] Louv R. Last Child in the Woods [website]. Available: http://richardlouv.com/books/last-child/ [accessed 22 September 2015]

[2] Egan T. Nature-deficit disorder. The New York Times, The Opinion Pages section, The Opinionator blog (29 March 2012). Available: http://opinionator.blogs.nytimes.com/2012/03/29/nature-deficit-disorder/ [accessed 22 September 2015]

[3] Todd M. Is our disconnect from nature a disorder? Pacific Standard, Nature & Technology section (5 April 2013). Available: http://www.psmag.com/nature-and-technology/nature-deficit-disorder-outdoors-outside-54707 [accessed 22 September 2015]

[4] Lenhart A. Teens, Social Media & Technology Overview 2015. Washington, DC:Pew Research Center (2015). Available: http://www.pewinternet.org/2015/04/09/teens-social-media-technology-2015/ [accessed 22 September 2015]

[5] Mainella FP Outdoor-based play and reconnection to nature: a neglected pathway to positive youth development. New Dir Youth Dev 2011 130 89 104 2011; 10.1002/yd.399 21786412

[6] CDC. Adolescent and School Health: Physical Activity Facts [website]. Atlanta, GA:U.S. Centers for Disease Control and Prevention (updated 19 May 2015). Available: http://www.cdc.gov/healthyyouth/physicalactivity/facts.htm [accessed 22 September 2015]

[7] CDC. Adolescent and School Health: Childhood Obesity Facts [website]. Atlanta, GA:U.S. Centers for Disease Control and Prevention (updated 24 April 2015). Available: http://www.cdc.gov/healthyyouth/obesity/facts.htm [accessed 22 September 2015]

[8] Kuo FE (Ming). Parks and Other Green Environments: Essential Components of a Healthy Human Habitat. Ashburn, VA:National Recreation and Park Association (2010). Available: http://www.nrpa.org/uploadedFiles/nrpa.org/Publications_and_Research/Research/Papers/MingKuo-Research-Paper.pdf [accessed 22 September 2015]

[9] James P A review of the health benefits of greenness. Curr Epidemiol Rep 2 2 131 142 2015; 10.1007/s40471-015-0043-7 26185745 PMC4500194

[10] Gentry BS, et al. Improving Human Health by Increasing Access to Natural Areas: Linking Research to Action at Scale. Report of the 2014 Berkley Workshop (Gentry BS, ed.). New Haven, CT:Yale School of Forestry and Environmental Studies (2015). Available: http://environment.yale.edu/publication-series/6132.html [accessed 22 September 2015]

[11] Chawla L Policy statement #20137: improving health and wellness through access to nature. Child Youth Environ 24 1 196 197 2014; 10.7721/chilyoutenvi.24.1.0196

[12] Nieuwenhuijsen MJ Positive health effects of the natural outdoor environment in typical populations in different regions in Europe (PHENOTYPE): a study programme protocol. BMJ Open 4 4 e004951 2014; 10.1136/bmjopen-2014-004951 PMC3996820 24740979

[13] Townsend M, et al. Healthy Parks Healthy People: the state of the evidence 2015. Victoria, AU:Parks Victoria (July 2015). Available: http://www.hphpcentral.com/wp-content/uploads/2015/07/HPHP_state-of-the-evidence_2015.pdf [accessed 22 September 2015]

[14] Swinburn BA The green prescription study: a randomized controlled trial of written exercise advice provided by general practitioners. Am J Public Health 88 2 288 291 1998; 10.2105/AJPH.88.2.288 9491025 PMC1508188

[15] NPS. Healthy Parks Healthy People US [website]. Washington, DC:National Park Service, U.S. Department of the Interior (2015). Available: http://www.nps.gov/public_health/hp/hphp.htm [accessed 22 September 2015]

[16] DOI. Every Kid in a Park [website]. Washington, DC:U.S. Department of the Interior. Available: https://everykidinapark.gov/ [accessed 22 September 2015]

[17] McCurdy LE Using nature and outdoor activity to improve children’s health. Curr Probl Pediatr Adolesc Health Care 40 5 102 117 2010; 10.1016/j.cppeds.2010.02.003 20381783

[18] Dadvand P Risks and benefits of green spaces for children: a cross-sectional study of associations with sedentary behavior, obesity, asthma, and allergy. Environ Health Perspect 122 12 1329 1339 2014; 10.1289/ehp.1308038 25157960 PMC4256701

[19] Thompson Coon J Does participating in physical activity in outdoor natural environments have a greater effect on physical and mental wellbeing than physical activity indoors? A systematic review. Environ Sci Technol 45 5 1761 1762 2011; 10.1021/es102947t 21291246

[20] Pasanen TP The relationship between perceived health and physical activity indoors, outdoors in built environments, and outdoors in nature. Appl Psychol Health Well-Being 6 3 324 346 2014; 10.1111/aphw.12031 25044598 PMC4233975

[21] Wu C-D Linking student performance in Massachusetts elementary schools with the “greenness” of school surroundings using remote sensing. PLoS ONE 9 10 e108548 2014; 10.1371/journal.pone.0108548 25310542 PMC4195655

[22] Taylor AF Views of nature and self-discipline: evidence from inner city children. J Environ Psychol 22 1–2 49 63 2002; 10.1006/jevp.2001.0241

[23] Dadvand P Green spaces and cognitive development in primary schoolchildren. Proc Natl Acad Sci USA 112 26 7937 7942 2015; 10.1073/pnas.1503402112 26080420 PMC4491800

[24] Wells NM At home with nature: effects of “greenness” on children’s cognitive functioning. Environ Behav 32 6 775 795 2000; 10.1177/00139160021972793

[25] Taylor AF Kuo FE (Ming) Could exposure to everyday green spaces help treat ADHD? Evidence from children’s play settings. Appl Psychol Health Well-Being 3 3 281 303 2011; 10.1111/j.1758-0854.2011.01052.x

[26] Kuo FE (Ming) Taylor AF A potential natural treatment for attention-deficit/hyperactivity disorder: evidence from a national study. Am J Public Health 94 9 1580 1586 2004; PMID:15333318 10.2105/ajph.94.9.1580 PMC1448497

[27] Taylor AF Kuo FE (Ming) Children with attention deficits concentrate better after walk in the park. J Atten Disord 12 5 402 409 2009; 10.1177/1087054708323000 18725656

[28] Amoly E Green and blue spaces and behavioral development in Barcelona schoolchildren: the BREATHE Project. Environ Health Perspect 122 12 1351 1358 2014; 10.1289/ehp.1408215 25204008 PMC4256702

[29] He M Effect of time spent outdoors at school on the development of myopia among children in China: a randomized clinical trial. JAMA 314 11 1142 1148 2015; 10.1001/jama.2015.10803 26372583

[30] Wu P-C Outdoor activity during class recess reduces myopia onset and progression in school children. Ophthalmology 120 5 1080 1085 2013; 10.1016/j.ophtha.2012.11.009 23462271

[31] Sherwin JC The association between time spent outdoors and myopia in children and adolescents. Ophthalmology 119 10 2141 2151 2012; 10.1016/j.ophtha.2012.04.020 22809757

[32] Rose KA Outdoor activity reduces the prevalence of myopia in children. Ophthalmology 115 8 1279 1285 2008; 10.1016/j.ophtha.2007.12.019 18294691

[33] Mitchell R Popham F Effect of exposure to natural environment on health inequalities: an observational population study. Lancet 372 9650 1655 1660 2008; 10.1016/S0140-6736(08)61689-X 18994663

[34] Dadvand P Surrounding greenness and exposure to air pollution during pregnancy: an analysis of personal monitoring data. Environ Health Perspect 120 9 1286 1290 2012; 10.1289/ehp.1104609 22647671 PMC3440116

[35] Gidlöf-Gunnarsson A Öhrström E Noise and well-being in urban residential environments: the potential role of perceived availability to nearby green areas. Landsc Urban Plann 83 2 115 126 2007; 10.1016/j.landurbplan.2007.03.003

[36] Borris M Simulating future trends in urban stormwater quality for changing climate, urban land use and environmental controls. Water Sci Technol 68 9 2082 2089 2013; 10.2166/wst.2013.465 24225112

[37] Bowler DE Urban greening to cool towns and cities: a systematic review of the empirical evidence. Landsc Urban Plann 97 3 147 155 2010; 10.1016/j.landurbplan.2010.05.006

[38] Institute at the Golden Gate. Park Prescriptions: Profiles and Resources for Good Health from the Great Outdoors. Sausalito,CA:Institute at the Golden Gate (2010). Available: http://www.americantrails.org/resources/health/Park-Prescriptions-Health-Great-Outdoors.html [accessed 22 September 2015]

[39] John Hancock. John Hancock Vitality Program [website]. Boston, MA, and Valhalla, NY:John Hancock Life Insurance Company. Available: https://www.johnhancockinsurance.com/life/John-Hancock-Vitality-Program.aspx [accessed 22 September 2015]

[40] Oscar [website]. New Nork NY: Oscar Insurance Company. Available: https://www.hioscar.com/ [accessed 22 September 2015]

[41] NRPA. Park Prescriptions [website]. Ashburn, VA:National Recreation and Park Association (2015). Available: http://www.nrpa.org/Grants-and-Partners/Recreation-and-Health/Park-Prescriptions/ [accessed 22 September 2015]

[42] Razani N Healing through nature: a park-based health intervention for young people in Oakland, California. Child Youth Environ 25 1 147 159 2015; 10.7721/chilyoutenvi.25.1.0147

[43] EBRPD. Park Prescriptions: Stay Healthy in Nature Everyday [website]. Oakland, CA:East Bay Regional Park District (2015). Available: http://www.ebparks.org/activities/hphp/shine [accessed 22 September 2015]

[44] Fullilove MT (1998). Promoting social cohesion to improve health.. J Am Med Womens Assoc.

[45] Triguero-Mas M Natural outdoor environments and mental and physical health: relationships and mechanisms. Environ Int 77 35 41 2015; 10.1016/j.envint.2015.01.012 25638643

[46] Kaplan R, Kaplan S. The Experience of Nature: A Psychological Perspective. Cambridge, United Kingdom:Cambridge University Press (1989)

[47] Kuo M How might contact with nature promote human health? Promising mechanisms and a possible central pathway. Front Psychol 6 1093 2015; 10.3389/fpsyg.2015.01093 26379564 PMC4548093

[48] Wells NM, Donofrio GA. Urban planning, the natural environment, and public health. In: Encyclopedia of Environmental Health, Vol. 5 (Nriagu JO, ed.). Burlington, MA:Elsevier (2011)

[49] Almanza E A study of community design, greenness, and physical activity in children using satellite, GPS and accelerometer data. Health Place 18 1 46 54 2012; 10.1016/j.healthplace.2011.09.003 22243906 PMC3399710

[50] McCormack GR Physical activity patterns in urban neighborhood parks: insights from a multiple case study. BMC Public Health 14 962 2014; 10.1186/1471-2458-14-962 25230763 PMC4247115

[51] McCormack GR Characteristics of urban parks associated with park use and physical activity: a review of qualitative research. Health Pace 16 4 712 726 2010; 10.1016/j.healthplace.2010.03.003 20356780

[52] Bancroft C Association of proximity and density of parks and objectively measured physical activity in the United States: a systematic review. Soc Sci Med 138 22 30 2015; 10.1016/j.socscimed.2015.05.034 26043433

[53] Cohen DA Impact of park renovations on park use and park-based physical activity. J Phys Act Health 12 2 289 295 2015; 10.1123/jpah.2013-0165 24956608 PMC4851467

[54] Cohen DA The potential for pocket parks to increase physical activity. Am J Health Promot 28 suppl 3 S19 S26 2014; 10.4278/ajhp.130430-QUAN-213 24380461 PMC4091959

[55] Han B How much neighborhood parks contribute to local residents’ physical activity in the City of Los Angeles: a meta-analysis. Prev Med 69 suppl 1 S106 110 2014; 10.1016/j.ypmed.2014.08.033 25199733 PMC4268157

[56] Rader NE We never see children in parks: a qualitative examination of the role of safety concerns on physical activity among children. J Phys Act Health; 10.1123/jpah.2014-0053 [in press] 25156307

[57] Institute at the Golden Gate. Healthy Parks, Healthy People [website]. Sausalito, CA:Institute at the Golden Gate. Available: http://instituteatgoldengate.org/health [accessed 22 September 2015]

[58] EBRPD. Healthy Parks Healthy People Bay Area [website]. Oakland, CA:East Bay Regional Parks District. Available: http://www.ebparks.org/activities/hphp/HPHPBayArea [accessed 22 September 2015]

[59] SF Rec & Park. Healthy Parks, Healthy People [website]. San Francisco, CA:San Francisco Recreation and Parks (2015). Available: http://sfrecpark.org/recprogram/healthy-parks-healthy-people [accessed 22 September 2015]

[60] DC AAP. Chapter Initiatives: DC Park Rx [website]. Washington, DC:American Academy of Pediatrics, District of Columbia Chapter (2015). Available: http://aapdc.org/chapter-initiatives/dc-park-rx/ [accessed 22 September 2015]

[61] Astell-Burt T Do low-income neighborhoods have the least green space? A cross-sectional study of Australia’s most populous cities. BMC Public Health 14 292 2014; 10.1186/1471-2458-14-292 24678610 PMC4005631

[62] Ralston R, et al. The role of Informationists in Delivering Geospatial Intelligence to Healthcare Professionals [presentation]. Available: https://scholarworks.iupui.edu/bitstream/handle/1805/5955/ralston-2013-the-role.pdf?sequence=1 [accessed 22 September 2015]

[63] New Mexico Health Care Takes on Diabetes. Welcome to Prescription Trails in New Mexico [website]. Albuquerque, NM:New Mexico Health Care Takes on Diabetes (2015). Available: http://prescriptiontrails.org/index/index.shtml [accessed 22 September 2015]

[64] Spence C. Taking Park Prescriptions Nationwide [website]. Sausalito, CA:Institute at the Golden Gate (updated 28 October 2014). Available: http://instituteatgoldengate.org/blog/taking-park-prescriptions-nationwide?context=tag-parkrx [accessed 22 September 2015]

[65] Dadvand P, et al. Green spaces and health. In: Abstracts of the 2015 Conference of the International Society of Environmental Epidemiology (ISEE). Abstract 396. Research Triangle Park, NC:Environmental Health Perspectives; 10.1289/ehp.isee2015

[66] GWA Park Rx. Park Rx Greater Williamsburg Area [website]. Williamsburg, VA:Park Rx Greater Williamsburg Area (2015). Available: http://www.gwaparkrx.com/ [accessed 22 September 2015]

[67] Kaiser Permanente. About Kaiser Permanente [website]. Oakland, CA:Kaiser Permanente (2015). Available: http://share.kaiserpermanente.org/about-kaiser-permanente [accessed 22 September 2015]

[68] Frumkin H Beyond toxicity: human health and the natural environment. Am J Prev Med 20 3 234 240 2001; 10.1016/S0749-3797(00)00317-2 11275453

